# Bis(2,6-dihy­droxy­benzoato-κ^2^ *O* ^1^,*O* ^1′^)(nitrato-κ^2^ *O*,*O*′)bis­(1,10-phenanthroline-κ^2^ *N*,*N*′)neodymium(III)

**DOI:** 10.1107/S1600536810042583

**Published:** 2010-10-30

**Authors:** Junjia Zheng, Hongxiao Jin, Hongliang Ge

**Affiliations:** a College of Materials Science and Engineering, China Jiliang University, Hangzhou 310018, People’s Republic of China

## Abstract

In the mononuclear title complex, [Nd(C_7_H_5_O_4_)_2_(NO_3_)(C_12_H_8_N_2_)_2_], the Nd^III^ atom is in a distorted bicapped square-anti­prismatic geometry formed by four N atoms from two chelating 1,10-phenanthroline (phen) ligands, four O atoms from two 2,6-dihy­droxy­benzoate (DHB) ligands and two O atoms from a nitrate anion. π–π stacking inter­actions between the phen and DHB ligands of adjacent complexes [centroid–centroid distances = 3.520 (6) and 3.798 (6) Å] stabilize the crystal structure. Intra­molecular O—H⋯O hydrogen bonds are observed in the DHB ligands.

## Related literature

For applications of rare earth complexes, see: de Sa *et al.* (2000 ▶). For related structures, see: Ma *et al.* (2010 ▶); Yang *et al.* (2008 ▶).
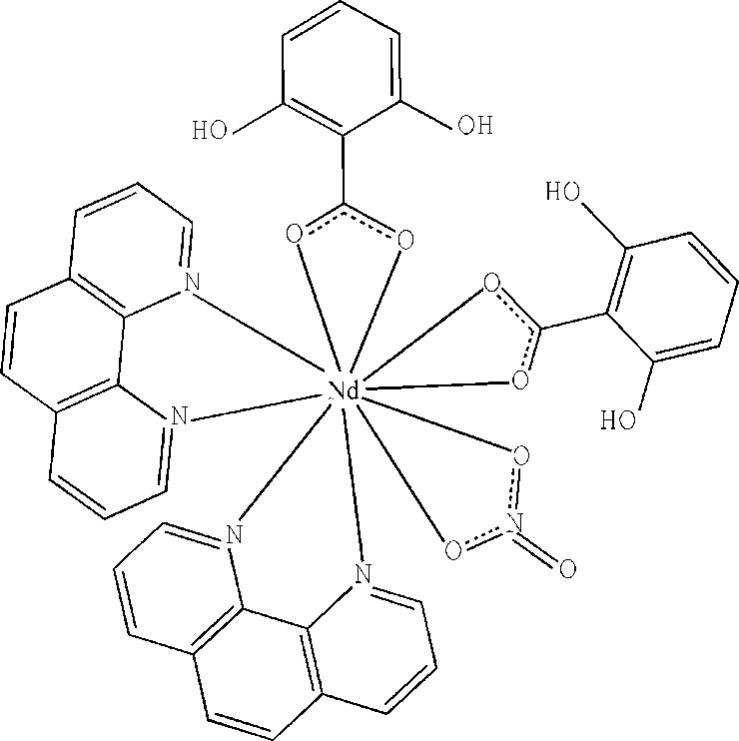


## Experimental

### 

#### Crystal data

[Nd(C_7_H_5_O_4_)_2_(NO_3_)(C_12_H_8_N_2_)_2_]
*M* *_r_* = 872.89
Monoclinic, 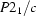

*a* = 11.2359 (2) Å
*b* = 26.7878 (5) Å
*c* = 14.3710 (4) Å
β = 127.790 (2)°
*V* = 3418.24 (16) Å^3^
*Z* = 4
Mo *K*α radiation
μ = 1.59 mm^−1^
*T* = 298 K
0.28 × 0.21 × 0.15 mm

#### Data collection

Oxford Diffraction Gemini S Ultra CCD diffractometer
Absorption correction: multi-scan (*CrysAlis PRO*; Oxford Diffraction, 2006 ▶) *T*_min_ = 0.664, *T*_max_ = 0.796
18058 measured reflections
6139 independent reflections
4859 reflections with *I* > 2σ(*I*)
*R*_int_ = 0.023

#### Refinement

*R*[*F* ^2^ > 2σ(*F* ^2^)] = 0.023
*wR*(*F* ^2^) = 0.049
*S* = 0.95
6139 reflections
496 parameters
H-atom parameters constrained
Δρ_max_ = 0.38 e Å^−3^
Δρ_min_ = −0.37 e Å^−3^

### 

Data collection: *CrysAlis PRO* (Oxford Diffraction, 2006 ▶); cell refinement: *CrysAlis PRO*; data reduction: *CrysAlis PRO*; program(s) used to solve structure: *SHELXS97* (Sheldrick, 2008 ▶); program(s) used to refine structure: *SHELXL97* (Sheldrick, 2008 ▶); molecular graphics: *ORTEP-3* (Farrugia, 1997 ▶) and *DIAMOND* (Brandenburg & Berndt, 1999 ▶); software used to prepare material for publication: *SHELXL97*.

## Supplementary Material

Crystal structure: contains datablocks I, global. DOI: 10.1107/S1600536810042583/hy2364sup1.cif

Structure factors: contains datablocks I. DOI: 10.1107/S1600536810042583/hy2364Isup2.hkl

Additional supplementary materials:  crystallographic information; 3D view; checkCIF report

## Figures and Tables

**Table 1:** Selected bond lengths (Å)

Nd1—O1	2.5256 (17)
Nd1—O2	2.5991 (16)
Nd1—O5	2.5297 (18)
Nd1—O6	2.5325 (17)
Nd1—O9	2.6060 (17)
Nd1—O10	2.5573 (17)
Nd1—N1	2.673 (2)
Nd1—N2	2.651 (2)
Nd1—N3	2.628 (2)
Nd1—N4	2.603 (2)

**Table 2:** Hydrogen-bond geometry (Å, °)

*D*—H⋯*A*	*D*—H	H⋯*A*	*D*⋯*A*	*D*—H⋯*A*
O3—H31⋯O1	0.82	1.86	2.581 (2)	147
O4—H27⋯O2	0.82	1.87	2.582 (3)	145
O7—H34⋯O6	0.82	1.87	2.592 (2)	146
O8—H38⋯O5	0.82	1.83	2.562 (3)	148

**Fig. 1.**
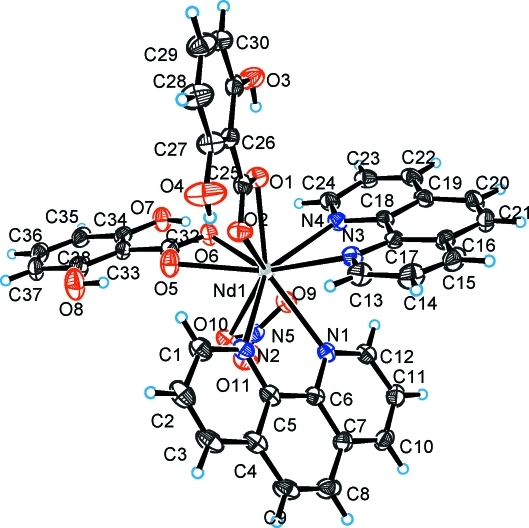
The molecular structure of the title compound. Displacement ellipsoids are drawn at the 30% probability level.

**Fig. 2.**
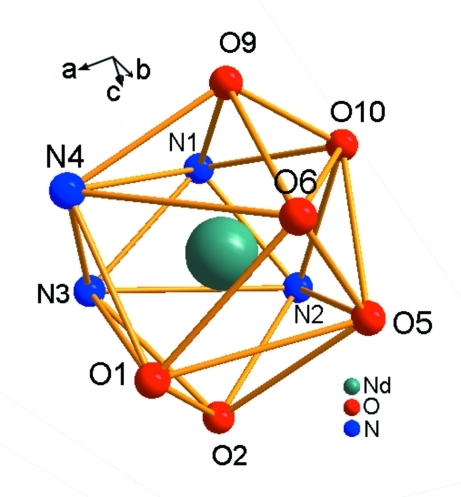
Coordination geometry around Nd.

**Fig. 3.**
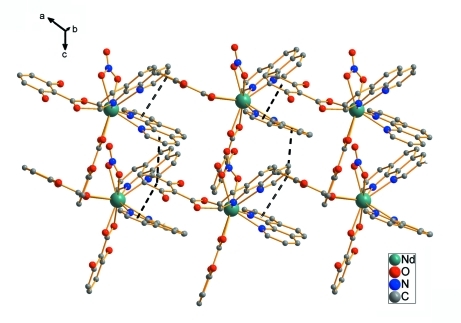
Crystal packing of the title compound. Dashed lines denote π–π stacking interactions.

## supplementary crystallographic information

### Comment

Luminescent materials based on lanthanide complexes have been extensively studied owing to their unique structures and potential applications as luminescent polymer films, active optical fibers for data transmission and light-emitting devices (Ma *et al.*, 2010; Sa *et al.*, 2000; Yang *et al.*, 2008). Herein, the title compound is synthesized and its crystal structure is reported.

The mononuclear structure of the title compound is shown in Fig. 1. The ten-coordinated geometry of the Nd^III^ ion is completed by four 2,6-dihydroxybenzoate (DHB) O atoms, four phenanthroline N atoms and two nitrate O atoms. The complex forms a ten-coordinated distorted bicapped square antiprismatic structure (Fig. 2), in which the set of O6, O10, N4 and N1 and the set of O1, O5, N2 and N3 form two approximate square planes, respectively. The ninth coordinate atom O2 and tenth coordinate atom O9 are located above and under the two planes at the bicapped positions. The O2—Nd1–O9 bond angle is 175.69 (6)° close to 180°, which indicates the O2, Nd1 and O9 lying almost on a line and being regarded as an axis. Because the coordinate O2 and O9 atoms are excluded by O6, O10, N4 and N1 (formed the above plane) and O1, O5, N2 and N3 (formed the under plane), respectively, the bond distances of Nd1—O2 [2.5991 (16) Å] and Nd1—O9 [2.6060 (17) Å] are consumedly longer than the ones of Nd1—O1 [2.5256 (17) Å] and Nd1—O10 [2.5573 (17) Å].

In the crystal structure, π–π stacking interactions between the pyridine ring of phen and the benzene ring of DHB link the discrete mononuclear complexes into a two-dimensional layer (Fig. 3). The centroid–centroid distances between the rings are 3.520 (6) and 3.798 (6) Å, which obviously helps to stabilize the structure.

### Experimental

All reagents are commercially available and of analytical grade. NdNO_3_.6H_2_O (0.219 g, 0.5 mmol), 2,6-dihydroxybenzoic acid (0.074 g, 0.5 mmol),1,10-phenanthroline (0.090 g, 0.5 mmol) and NaHCO_3_ (0.042 g, 0.5 mmol) were dissolved in water–ethanol solution (10 ml, 1:1). The solution was refluxed for 4 h, and filtered after cooling to room temperature. Orange single crystals were obtained from the filtrate after 2 d.

### Refinement

H atoms were positioned geometrically and refined as riding atoms, with C—H = 0.93 and O—H = 0.82 Å and with *U*_iso_(H) = 1.2(1.5 for hydroxy)*U*_eq_(C,O).

### Crystal data

**Table:**

[Nd(C_7_H_5_O_4_)_2_(NO_3_)(C_12_H_8_N_2_)_2_]	*F*(000) = 1748
*M**_r_* = 872.89	*D*_x_ = 1.696 Mg m^−^^3^
Monoclinic, *P*2_1_/*c*	Mo *K*α radiation, λ = 0.71073 Å
Hall symbol: -P 2ybc	Cell parameters from 11150 reflections
*a* = 11.2359 (2) Å	θ = 2.9–29.1°
*b* = 26.7878 (5) Å	µ = 1.59 mm^−^^1^
*c* = 14.3710 (4) Å	*T* = 298 K
β = 127.790 (2)°	Block, orange
*V* = 3418.24 (16) Å^3^	0.28 × 0.21 × 0.15 mm
*Z* = 4	

### Data collection

**Table:**

Oxford Diffraction Gemini S Ultra CCD diffractometer	6139 independent reflections
Radiation source: Enhance (Mo) X-ray Source	4859 reflections with *I* > 2σ(*I*)
graphite	*R*_int_ = 0.023
Detector resolution: 15.9149 pixels mm^-1^	θ_max_ = 25.2°, θ_min_ = 2.9°
ω scans	*h* = −13→13
Absorption correction: multi-scan (*CrysAlis PRO*; Oxford Diffraction, 2006)	*k* = −32→31
*T*_min_ = 0.664, *T*_max_ = 0.796	*l* = −17→15
18058 measured reflections	

### Refinement

**Table:**

Refinement on *F*^2^	Primary atom site location: structure-invariant direct methods
Least-squares matrix: full	Secondary atom site location: difference Fourier map
*R*[*F*^2^ > 2σ(*F*^2^)] = 0.023	Hydrogen site location: inferred from neighbouring sites
*wR*(*F*^2^) = 0.049	H-atom parameters constrained
*S* = 0.95	*w* = 1/[σ^2^(*F*_o_^2^) + (0.026*P*)^2^] where *P* = (*F*_o_^2^ + 2*F*_c_^2^)/3
6139 reflections	(Δ/σ)_max_ = 0.001
496 parameters	Δρ_max_ = 0.38 e Å^−^^3^
0 restraints	Δρ_min_ = −0.37 e Å^−^^3^

### Fractional atomic coordinates and isotropic or equivalent isotropic displacement parameters (Å^2^)

**Table:**

	*x*	*y*	*z*	*U*_iso_*/*U*_eq_	
Nd1	0.068277 (15)	0.638621 (5)	0.280312 (12)	0.03184 (5)	
O1	0.1545 (2)	0.66194 (7)	0.48337 (15)	0.0445 (5)	
O2	0.1118 (2)	0.58179 (7)	0.44526 (15)	0.0458 (5)	
O3	0.2418 (2)	0.69710 (7)	0.68349 (17)	0.0597 (5)	
H31	0.2182	0.6979	0.6170	0.090*	
O4	0.1664 (3)	0.52057 (7)	0.60604 (19)	0.0828 (8)	
H27	0.1539	0.5288	0.5456	0.124*	
O5	0.3377 (2)	0.60803 (7)	0.40378 (17)	0.0521 (5)	
O6	0.3064 (2)	0.68871 (7)	0.36811 (16)	0.0441 (4)	
O7	0.5201 (2)	0.74239 (7)	0.39922 (17)	0.0535 (5)	
H34	0.4352	0.7365	0.3781	0.080*	
O8	0.5823 (3)	0.56507 (8)	0.4709 (2)	0.0737 (6)	
H38	0.4963	0.5674	0.4499	0.111*	
O9	0.0412 (2)	0.69978 (7)	0.12734 (16)	0.0471 (5)	
O10	0.1513 (2)	0.62994 (7)	0.15044 (18)	0.0525 (5)	
O11	0.1319 (3)	0.68609 (10)	0.03319 (19)	0.0831 (8)	
N1	−0.1692 (2)	0.61789 (8)	0.05894 (18)	0.0349 (5)	
N2	0.0231 (2)	0.54467 (7)	0.20773 (19)	0.0375 (5)	
N3	−0.1856 (2)	0.63390 (8)	0.25221 (18)	0.0381 (5)	
N4	−0.0620 (2)	0.72232 (8)	0.26242 (18)	0.0360 (5)	
N5	0.1082 (3)	0.67266 (10)	0.1013 (2)	0.0478 (6)	
C1	0.1163 (3)	0.50855 (10)	0.2787 (3)	0.0454 (7)	
H1	0.1914	0.5162	0.3575	0.055*	
C2	0.1072 (4)	0.45960 (10)	0.2408 (3)	0.0546 (8)	
H2	0.1749	0.4355	0.2938	0.066*	
C3	0.0000 (4)	0.44770 (11)	0.1275 (3)	0.0511 (8)	
H3	−0.0064	0.4153	0.1014	0.061*	
C4	−0.1016 (3)	0.48396 (10)	0.0488 (3)	0.0414 (7)	
C5	−0.0854 (3)	0.53267 (9)	0.0931 (2)	0.0351 (6)	
C6	−0.1877 (3)	0.57118 (9)	0.0147 (2)	0.0337 (6)	
C7	−0.3023 (3)	0.55995 (10)	−0.1047 (2)	0.0404 (7)	
C8	−0.3149 (4)	0.51012 (12)	−0.1458 (3)	0.0520 (8)	
H8	−0.3905	0.5025	−0.2245	0.062*	
C9	−0.2196 (4)	0.47427 (11)	−0.0729 (3)	0.0501 (8)	
H9	−0.2304	0.4422	−0.1021	0.060*	
C10	−0.3989 (3)	0.59882 (12)	−0.1773 (2)	0.0499 (8)	
H10	−0.4748	0.5930	−0.2569	0.060*	
C11	−0.3823 (3)	0.64493 (11)	−0.1319 (2)	0.0467 (7)	
H11	−0.4476	0.6707	−0.1790	0.056*	
C12	−0.2661 (3)	0.65268 (10)	−0.0140 (2)	0.0420 (7)	
H12	−0.2551	0.6845	0.0163	0.050*	
C13	−0.2459 (3)	0.59062 (11)	0.2495 (2)	0.0474 (7)	
H13	−0.1872	0.5619	0.2738	0.057*	
C14	−0.3934 (4)	0.58626 (13)	0.2119 (3)	0.0570 (8)	
H14	−0.4315	0.5553	0.2111	0.068*	
C15	−0.4805 (4)	0.62816 (13)	0.1762 (3)	0.0583 (9)	
H15	−0.5794	0.6257	0.1496	0.070*	
C16	−0.4216 (3)	0.67480 (11)	0.1796 (2)	0.0428 (7)	
C17	−0.2723 (3)	0.67573 (10)	0.2186 (2)	0.0358 (6)	
C18	−0.2042 (3)	0.72281 (9)	0.2286 (2)	0.0341 (6)	
C19	−0.2843 (3)	0.76743 (10)	0.2050 (2)	0.0424 (7)	
C20	−0.4380 (3)	0.76430 (12)	0.1606 (2)	0.0531 (8)	
H20	−0.4938	0.7935	0.1398	0.064*	
C21	−0.5034 (3)	0.72081 (13)	0.1482 (2)	0.0568 (8)	
H21	−0.6037	0.7202	0.1188	0.068*	
C22	−0.2088 (4)	0.81226 (11)	0.2244 (2)	0.0485 (7)	
H22	−0.2571	0.8425	0.2122	0.058*	
C23	−0.0669 (4)	0.81185 (10)	0.2605 (2)	0.0495 (7)	
H23	−0.0157	0.8416	0.2749	0.059*	
C24	0.0033 (3)	0.76606 (10)	0.2764 (2)	0.0436 (7)	
H24	0.0999	0.7662	0.2977	0.052*	
C25	0.1535 (3)	0.61801 (11)	0.5154 (2)	0.0372 (6)	
C26	0.2018 (3)	0.60945 (10)	0.6361 (2)	0.0355 (6)	
C27	0.2063 (4)	0.56095 (11)	0.6757 (3)	0.0512 (8)	
C28	0.2554 (4)	0.55331 (13)	0.7898 (3)	0.0643 (9)	
H28	0.2603	0.5212	0.8164	0.077*	
C29	0.2962 (4)	0.59309 (13)	0.8626 (3)	0.0605 (9)	
H29	0.3278	0.5876	0.9386	0.073*	
C30	0.2920 (3)	0.64062 (12)	0.8275 (3)	0.0543 (8)	
H30	0.3213	0.6671	0.8794	0.065*	
C31	0.2439 (3)	0.64953 (10)	0.7139 (2)	0.0406 (7)	
C32	0.3881 (3)	0.65023 (11)	0.4013 (2)	0.0411 (7)	
C33	0.5412 (3)	0.65354 (10)	0.4341 (2)	0.0386 (6)	
C34	0.5980 (3)	0.69969 (11)	0.4303 (2)	0.0418 (7)	
C35	0.7421 (3)	0.70261 (13)	0.4607 (2)	0.0510 (8)	
H35	0.7805	0.7330	0.4588	0.061*	
C36	0.8260 (3)	0.65970 (14)	0.4937 (2)	0.0580 (9)	
H36	0.9222	0.6618	0.5145	0.070*	
C37	0.7746 (3)	0.61407 (14)	0.4972 (3)	0.0584 (8)	
H37	0.8344	0.5858	0.5195	0.070*	
C38	0.6316 (3)	0.61079 (12)	0.4670 (2)	0.0515 (7)	

### Atomic displacement parameters (Å^2^)

**Table:**

	*U*^11^	*U*^22^	*U*^33^	*U*^12^	*U*^13^	*U*^23^
Nd1	0.03283 (8)	0.02688 (8)	0.03861 (8)	0.00037 (7)	0.02331 (7)	−0.00004 (7)
O1	0.0526 (12)	0.0346 (11)	0.0431 (11)	−0.0009 (10)	0.0278 (10)	0.0030 (10)
O2	0.0568 (13)	0.0393 (11)	0.0430 (11)	−0.0027 (10)	0.0314 (10)	−0.0018 (10)
O3	0.0674 (15)	0.0414 (13)	0.0479 (12)	−0.0016 (11)	0.0238 (11)	−0.0069 (11)
O4	0.146 (2)	0.0402 (13)	0.0605 (14)	−0.0189 (15)	0.0625 (16)	−0.0032 (12)
O5	0.0407 (12)	0.0417 (12)	0.0685 (13)	0.0049 (10)	0.0307 (10)	0.0136 (11)
O6	0.0354 (11)	0.0367 (11)	0.0588 (12)	0.0021 (9)	0.0282 (10)	−0.0007 (10)
O7	0.0435 (12)	0.0499 (13)	0.0683 (14)	0.0004 (10)	0.0349 (11)	0.0067 (11)
O8	0.0554 (14)	0.0518 (14)	0.1003 (18)	0.0167 (12)	0.0408 (14)	0.0153 (13)
O9	0.0530 (12)	0.0367 (11)	0.0562 (12)	0.0017 (10)	0.0359 (11)	0.0040 (10)
O10	0.0614 (14)	0.0442 (13)	0.0665 (13)	0.0032 (10)	0.0467 (12)	−0.0047 (11)
O11	0.0840 (18)	0.128 (2)	0.0584 (14)	−0.0030 (16)	0.0541 (14)	0.0151 (15)
N1	0.0368 (12)	0.0315 (12)	0.0400 (12)	0.0019 (11)	0.0253 (11)	0.0013 (11)
N2	0.0473 (14)	0.0268 (12)	0.0439 (14)	0.0031 (11)	0.0307 (12)	0.0037 (11)
N3	0.0405 (13)	0.0399 (14)	0.0403 (12)	−0.0058 (11)	0.0282 (11)	−0.0035 (11)
N4	0.0402 (13)	0.0298 (12)	0.0427 (12)	−0.0020 (10)	0.0278 (11)	−0.0025 (10)
N5	0.0425 (14)	0.0634 (18)	0.0385 (14)	−0.0123 (13)	0.0253 (12)	−0.0061 (13)
C1	0.0560 (19)	0.0344 (17)	0.0525 (18)	0.0059 (15)	0.0367 (16)	0.0062 (14)
C2	0.075 (2)	0.0324 (18)	0.077 (2)	0.0114 (16)	0.057 (2)	0.0138 (17)
C3	0.076 (2)	0.0260 (16)	0.078 (2)	−0.0052 (16)	0.061 (2)	−0.0055 (16)
C4	0.0520 (18)	0.0300 (16)	0.0636 (19)	−0.0086 (14)	0.0464 (17)	−0.0055 (15)
C5	0.0423 (16)	0.0284 (15)	0.0494 (17)	−0.0044 (13)	0.0356 (15)	−0.0007 (13)
C6	0.0356 (15)	0.0348 (16)	0.0417 (15)	−0.0079 (12)	0.0293 (13)	−0.0049 (13)
C7	0.0352 (15)	0.0464 (18)	0.0456 (17)	−0.0092 (14)	0.0278 (14)	−0.0082 (15)
C8	0.0499 (19)	0.055 (2)	0.0563 (19)	−0.0194 (17)	0.0352 (17)	−0.0196 (17)
C9	0.062 (2)	0.0365 (17)	0.072 (2)	−0.0207 (16)	0.0507 (19)	−0.0218 (17)
C10	0.0322 (16)	0.073 (2)	0.0395 (16)	−0.0069 (15)	0.0194 (14)	−0.0076 (16)
C11	0.0387 (16)	0.055 (2)	0.0450 (17)	0.0062 (15)	0.0249 (15)	0.0064 (15)
C12	0.0457 (17)	0.0394 (17)	0.0452 (17)	0.0054 (13)	0.0301 (15)	0.0009 (13)
C13	0.0514 (18)	0.0438 (18)	0.0537 (17)	−0.0093 (15)	0.0356 (16)	−0.0020 (15)
C14	0.054 (2)	0.061 (2)	0.063 (2)	−0.0262 (18)	0.0391 (18)	−0.0114 (18)
C15	0.0391 (17)	0.091 (3)	0.0480 (18)	−0.0172 (18)	0.0284 (15)	−0.0110 (18)
C16	0.0323 (15)	0.065 (2)	0.0313 (14)	−0.0046 (15)	0.0192 (13)	−0.0063 (14)
C17	0.0333 (15)	0.0470 (17)	0.0308 (14)	−0.0013 (13)	0.0216 (13)	−0.0031 (13)
C18	0.0371 (15)	0.0378 (16)	0.0298 (14)	0.0040 (13)	0.0216 (12)	0.0002 (12)
C19	0.0492 (18)	0.0476 (18)	0.0329 (15)	0.0141 (15)	0.0265 (14)	0.0038 (14)
C20	0.0500 (19)	0.063 (2)	0.0458 (18)	0.0210 (17)	0.0289 (16)	0.0048 (17)
C21	0.0353 (17)	0.091 (3)	0.0410 (17)	0.0132 (18)	0.0215 (15)	0.0016 (18)
C22	0.065 (2)	0.0362 (17)	0.0453 (17)	0.0148 (16)	0.0344 (16)	0.0031 (14)
C23	0.069 (2)	0.0297 (16)	0.0495 (17)	0.0005 (15)	0.0361 (17)	−0.0021 (14)
C24	0.0484 (17)	0.0334 (17)	0.0494 (17)	−0.0017 (14)	0.0301 (15)	−0.0045 (14)
C25	0.0294 (14)	0.0419 (17)	0.0405 (16)	−0.0018 (13)	0.0215 (13)	−0.0021 (14)
C26	0.0284 (14)	0.0373 (16)	0.0368 (15)	−0.0001 (12)	0.0179 (12)	0.0003 (13)
C27	0.062 (2)	0.0481 (19)	0.0456 (18)	−0.0102 (16)	0.0344 (16)	−0.0044 (16)
C28	0.086 (3)	0.060 (2)	0.050 (2)	−0.013 (2)	0.043 (2)	0.0065 (18)
C29	0.066 (2)	0.076 (2)	0.0423 (18)	−0.0125 (19)	0.0348 (17)	−0.0001 (19)
C30	0.0472 (18)	0.068 (2)	0.0433 (17)	−0.0009 (17)	0.0252 (15)	−0.0125 (17)
C31	0.0290 (14)	0.0433 (19)	0.0424 (17)	0.0017 (12)	0.0183 (13)	−0.0021 (14)
C32	0.0361 (16)	0.048 (2)	0.0367 (15)	−0.0019 (14)	0.0213 (13)	−0.0011 (13)
C33	0.0306 (14)	0.0460 (17)	0.0337 (15)	0.0067 (13)	0.0170 (13)	0.0034 (13)
C34	0.0353 (15)	0.0536 (19)	0.0322 (14)	−0.0008 (14)	0.0185 (13)	−0.0014 (14)
C35	0.0351 (17)	0.078 (2)	0.0401 (16)	−0.0053 (16)	0.0234 (14)	−0.0009 (16)
C36	0.0356 (17)	0.095 (3)	0.0448 (18)	0.0039 (19)	0.0253 (15)	−0.0009 (18)
C37	0.0397 (18)	0.078 (3)	0.0521 (18)	0.0230 (18)	0.0255 (16)	0.0069 (18)
C38	0.0462 (19)	0.055 (2)	0.0471 (17)	0.0083 (16)	0.0253 (15)	0.0053 (16)

### Geometric parameters (Å, °)

**Table:**

Nd1—O1	2.5256 (17)	C9—H9	0.9300
Nd1—O2	2.5991 (16)	C10—C11	1.355 (4)
Nd1—O5	2.5297 (18)	C10—H10	0.9300
Nd1—O6	2.5325 (17)	C11—C12	1.382 (4)
Nd1—O9	2.6060 (17)	C11—H11	0.9300
Nd1—O10	2.5573 (17)	C12—H12	0.9300
Nd1—N1	2.673 (2)	C13—C14	1.397 (4)
Nd1—N2	2.651 (2)	C13—H13	0.9300
Nd1—N3	2.628 (2)	C14—C15	1.366 (4)
Nd1—N4	2.603 (2)	C14—H14	0.9300
O1—C25	1.266 (3)	C15—C16	1.401 (4)
O2—C25	1.264 (3)	C15—H15	0.9300
O3—C31	1.343 (3)	C16—C17	1.406 (3)
O3—H31	0.8200	C16—C21	1.435 (4)
O4—C27	1.350 (3)	C17—C18	1.436 (3)
O4—H27	0.8200	C18—C19	1.406 (3)
O5—C32	1.275 (3)	C19—C22	1.396 (4)
O6—C32	1.264 (3)	C19—C20	1.431 (4)
O7—C34	1.340 (3)	C20—C21	1.330 (4)
O7—H34	0.8200	C20—H20	0.9300
O8—C38	1.359 (3)	C21—H21	0.9300
O8—H38	0.8200	C22—C23	1.341 (4)
O9—N5	1.257 (3)	C22—H22	0.9300
O10—N5	1.274 (3)	C23—C24	1.399 (4)
O11—N5	1.215 (3)	C23—H23	0.9300
N1—C12	1.324 (3)	C24—H24	0.9300
N1—C6	1.360 (3)	C25—C26	1.484 (3)
N2—C1	1.328 (3)	C26—C27	1.407 (4)
N2—C5	1.357 (3)	C26—C31	1.407 (3)
N3—C13	1.332 (3)	C27—C28	1.388 (4)
N3—C17	1.364 (3)	C28—C29	1.361 (4)
N4—C24	1.331 (3)	C28—H28	0.9300
N4—C18	1.356 (3)	C29—C30	1.359 (4)
C1—C2	1.399 (4)	C29—H29	0.9300
C1—H1	0.9300	C30—C31	1.390 (4)
C2—C3	1.342 (4)	C30—H30	0.9300
C2—H2	0.9300	C32—C33	1.481 (4)
C3—C4	1.394 (4)	C33—C38	1.406 (4)
C3—H3	0.9300	C33—C34	1.408 (4)
C4—C5	1.414 (3)	C34—C35	1.396 (4)
C4—C9	1.429 (4)	C35—C36	1.374 (4)
C5—C6	1.438 (3)	C35—H35	0.9300
C6—C7	1.409 (4)	C36—C37	1.366 (4)
C7—C10	1.401 (4)	C36—H36	0.9300
C7—C8	1.431 (4)	C37—C38	1.387 (4)
C8—C9	1.339 (4)	C37—H37	0.9300
C8—H8	0.9300		
			
O1—Nd1—O5	79.78 (6)	N1—C6—C7	121.9 (2)
O1—Nd1—O6	75.70 (6)	N1—C6—C5	118.3 (2)
O5—Nd1—O6	51.50 (6)	C7—C6—C5	119.8 (2)
O1—Nd1—O10	144.32 (6)	C10—C7—C6	117.5 (2)
O5—Nd1—O10	70.60 (6)	C10—C7—C8	123.4 (3)
O6—Nd1—O10	70.45 (6)	C6—C7—C8	119.1 (3)
O1—Nd1—O2	50.70 (6)	C9—C8—C7	121.1 (3)
O5—Nd1—O2	72.24 (6)	C9—C8—H8	119.4
O6—Nd1—O2	107.76 (6)	C7—C8—H8	119.4
O10—Nd1—O2	131.55 (6)	C8—C9—C4	121.6 (3)
O1—Nd1—N4	72.27 (6)	C8—C9—H9	119.2
O5—Nd1—N4	135.00 (7)	C4—C9—H9	119.2
O6—Nd1—N4	87.22 (6)	C11—C10—C7	120.2 (3)
O10—Nd1—N4	116.16 (6)	C11—C10—H10	119.9
O2—Nd1—N4	112.01 (6)	C7—C10—H10	119.9
O1—Nd1—O9	125.66 (6)	C10—C11—C12	118.4 (3)
O5—Nd1—O9	105.44 (6)	C10—C11—H11	120.8
O6—Nd1—O9	68.20 (6)	C12—C11—H11	120.8
O10—Nd1—O9	49.28 (6)	N1—C12—C11	124.4 (3)
O2—Nd1—O9	175.69 (6)	N1—C12—H12	117.8
N4—Nd1—O9	66.89 (6)	C11—C12—H12	117.8
O1—Nd1—N3	78.54 (6)	N3—C13—C14	123.1 (3)
O5—Nd1—N3	144.28 (6)	N3—C13—H13	118.5
O6—Nd1—N3	144.89 (6)	C14—C13—H13	118.5
O10—Nd1—N3	136.93 (7)	C15—C14—C13	119.0 (3)
O2—Nd1—N3	72.06 (6)	C15—C14—H14	120.5
N4—Nd1—N3	62.24 (6)	C13—C14—H14	120.5
O9—Nd1—N3	110.26 (6)	C14—C15—C16	120.3 (3)
O1—Nd1—N2	122.32 (6)	C14—C15—H15	119.8
O5—Nd1—N2	80.03 (7)	C16—C15—H15	119.8
O6—Nd1—N2	126.03 (6)	C15—C16—C17	116.9 (3)
O10—Nd1—N2	72.20 (6)	C15—C16—C21	123.8 (3)
O2—Nd1—N2	71.72 (6)	C17—C16—C21	119.2 (3)
N4—Nd1—N2	144.90 (7)	N3—C17—C16	123.0 (2)
O9—Nd1—N2	111.70 (6)	N3—C17—C18	117.7 (2)
N3—Nd1—N2	88.03 (6)	C16—C17—C18	119.2 (2)
O1—Nd1—N1	145.39 (6)	N4—C18—C19	122.2 (2)
O5—Nd1—N1	131.30 (6)	N4—C18—C17	117.7 (2)
O6—Nd1—N1	133.02 (6)	C19—C18—C17	120.0 (2)
O10—Nd1—N1	69.93 (6)	C22—C19—C18	117.5 (3)
O2—Nd1—N1	117.17 (6)	C22—C19—C20	124.0 (3)
N4—Nd1—N1	88.32 (6)	C18—C19—C20	118.4 (3)
O9—Nd1—N1	67.12 (6)	C21—C20—C19	121.9 (3)
N3—Nd1—N1	67.01 (6)	C21—C20—H20	119.1
N2—Nd1—N1	61.55 (7)	C19—C20—H20	119.1
O1—Nd1—C32	79.81 (6)	C20—C21—C16	121.0 (3)
O5—Nd1—C32	26.09 (6)	C20—C21—H21	119.5
O6—Nd1—C32	25.84 (6)	C16—C21—H21	119.5
O10—Nd1—C32	64.82 (7)	C23—C22—C19	120.2 (3)
O2—Nd1—C32	92.54 (7)	C23—C22—H22	119.9
N4—Nd1—C32	112.52 (7)	C19—C22—H22	119.9
O9—Nd1—C32	84.22 (7)	C22—C23—C24	119.2 (3)
N3—Nd1—C32	158.25 (7)	C22—C23—H23	120.4
N2—Nd1—C32	101.91 (7)	C24—C23—H23	120.4
N1—Nd1—C32	134.73 (6)	N4—C24—C23	123.0 (3)
O1—Nd1—C25	25.31 (7)	N4—C24—H24	118.5
O5—Nd1—C25	74.12 (6)	C23—C24—H24	118.5
O6—Nd1—C25	91.50 (6)	O2—C25—O1	120.3 (2)
O10—Nd1—C25	144.35 (7)	O2—C25—C26	120.3 (2)
O2—Nd1—C25	25.39 (6)	O1—C25—C26	119.4 (2)
N4—Nd1—C25	92.44 (7)	O2—C25—Nd1	61.82 (13)
O9—Nd1—C25	150.88 (7)	O1—C25—Nd1	58.49 (13)
N3—Nd1—C25	74.13 (6)	C26—C25—Nd1	177.36 (19)
N2—Nd1—C25	97.04 (7)	C27—C26—C31	118.2 (2)
N1—Nd1—C25	135.42 (6)	C27—C26—C25	120.8 (2)
C32—Nd1—C25	85.39 (7)	C31—C26—C25	121.0 (2)
C25—O1—Nd1	96.19 (15)	O4—C27—C28	118.0 (3)
C25—O2—Nd1	92.78 (15)	O4—C27—C26	121.8 (2)
C31—O3—H31	109.5	C28—C27—C26	120.2 (3)
C27—O4—H27	109.5	C29—C28—C27	119.7 (3)
C32—O5—Nd1	93.14 (16)	C29—C28—H28	120.1
C32—O6—Nd1	93.28 (15)	C27—C28—H28	120.1
C34—O7—H34	109.5	C30—C29—C28	122.0 (3)
C38—O8—H38	109.5	C30—C29—H29	119.0
N5—O9—Nd1	96.10 (15)	C28—C29—H29	119.0
N5—O10—Nd1	97.96 (13)	C29—C30—C31	119.8 (3)
C12—N1—C6	117.5 (2)	C29—C30—H30	120.1
C12—N1—Nd1	122.17 (17)	C31—C30—H30	120.1
C6—N1—Nd1	120.16 (16)	O3—C31—C30	117.5 (3)
C1—N2—C5	117.3 (2)	O3—C31—C26	122.3 (2)
C1—N2—Nd1	121.25 (17)	C30—C31—C26	120.1 (3)
C5—N2—Nd1	121.03 (16)	O6—C32—O5	120.1 (2)
C13—N3—C17	117.7 (2)	O6—C32—C33	120.3 (2)
C13—N3—Nd1	122.11 (18)	O5—C32—C33	119.6 (2)
C17—N3—Nd1	119.16 (15)	O6—C32—Nd1	60.87 (13)
C24—N4—C18	117.7 (2)	O5—C32—Nd1	60.78 (14)
C24—N4—Nd1	121.24 (17)	C33—C32—Nd1	165.83 (17)
C18—N4—Nd1	120.79 (16)	C38—C33—C34	118.4 (2)
O11—N5—O9	122.4 (3)	C38—C33—C32	121.1 (3)
O11—N5—O10	121.1 (3)	C34—C33—C32	120.4 (2)
O9—N5—O10	116.6 (2)	O7—C34—C35	116.9 (3)
N2—C1—C2	123.3 (3)	O7—C34—C33	122.9 (2)
N2—C1—H1	118.3	C35—C34—C33	120.2 (3)
C2—C1—H1	118.3	C36—C35—C34	118.7 (3)
C3—C2—C1	119.5 (3)	C36—C35—H35	120.7
C3—C2—H2	120.3	C34—C35—H35	120.7
C1—C2—H2	120.3	C37—C36—C35	123.0 (3)
C2—C3—C4	119.9 (3)	C37—C36—H36	118.5
C2—C3—H3	120.0	C35—C36—H36	118.5
C4—C3—H3	120.0	C36—C37—C38	118.7 (3)
C3—C4—C5	117.5 (3)	C36—C37—H37	120.7
C3—C4—C9	123.4 (3)	C38—C37—H37	120.7
C5—C4—C9	119.1 (3)	O8—C38—C37	117.9 (3)
N2—C5—C4	122.5 (3)	O8—C38—C33	121.3 (3)
N2—C5—C6	118.2 (2)	C37—C38—C33	120.9 (3)
C4—C5—C6	119.2 (2)		

### Hydrogen-bond geometry (Å, °)

**Table:**

*D*—H···*A*	*D*—H	H···*A*	*D*···*A*	*D*—H···*A*
O3—H31···O1	0.82	1.86	2.581 (2)	147
O4—H27···O2	0.82	1.87	2.582 (3)	145
O7—H34···O6	0.82	1.87	2.592 (2)	146
O8—H38···O5	0.82	1.83	2.562 (3)	148
